# L-carnosine Attenuates Bleomycin-Induced Oxidative Stress via NFκB Pathway in the Pathogenesis of Pulmonary Fibrosis

**DOI:** 10.3390/antiox11122462

**Published:** 2022-12-14

**Authors:** Jaehyun Park, Jimin Jang, Sang-Ryul Cha, Hyosin Baek, Jooyeon Lee, Seok-Ho Hong, Hyang-Ah Lee, Tae-Jin Lee, Se-Ran Yang

**Affiliations:** 1 Department of Thoracic and Cardiovascular Surgery, School of Medicine, Kangwon National University, Gangwondaehakgil l, Chuncheon 24341, Gangwon, Republic of Korea; 2 Department of Internal Medicine, School of Medicine, Kangwon National University, Gangwondaehakgil 1, Chuncheon 24341, Gangwon, Republic of Korea; 3 Department of Obstetrics and Gynecology, School of Medicine, Kangwon National University, Gangwondaehakgil 1, Chuncheon 24341, Gangwon, Republic of Korea; 4 Department of Bio-Health Convergence, Kangwon National University, Chuncheon 24341, Gangwon, Republic of Korea

**Keywords:** IPF, pulmonary fibrosis, L-carnosine, cigarette smoke, bleomycin, alveolar epithelial cells, ROS, apoptosis, fibroblast

## Abstract

Idiopathic Pulmonary fibrosis (IPF), a chronic interstitial lung disease, has pulmonary manifestations clinically characterized by collagen deposition, epithelial cell injury, and a decline in lung function. L-carnosine, a dipeptide consisting of β-alanine and L-histidine, has demonstrated a therapeutic effect on various diseases because of its pivotal function. Despite the effect of L-carnosine in experimental IPF mice, its anti-oxidative effect and associated intercellular pathway, particularly alveolar epithelial cells, remain unknown. Therefore, we demonstrated the anti-fibrotic and anti-inflammatory effects of L-carnosine via Reactive oxygen species (ROS) regulation in bleomycin (BLM)-induced IPF mice. The mice were intratracheally injected with BLM (3 mg/kg) and L-carnosine (150 mg/kg) was orally administrated for 2 weeks. BLM exposure increased the protein level of Nox2, Nox4, p53, and Caspase-3, whereas L-carnosine treatment suppressed the protein level of Nox2, Nox4, p53, and Caspase-3 cleavage in mice. In addition, the total SOD activity and mRNA level of *Sod2*, *catalase*, and *Nqo1* increased in mice treated with L-carnosine. At the cellular level, a human fibroblast (MRC-5) and mouse alveolar epithelial cell (MLE-12) were exposed to TGFβ1 following L-carnosine treatment to induce fibrogenesis. Moreover, MLE-12 cells were exposed to cigarette smoke extract (CSE). Consequently, L-carnosine treatment ameliorated fibrogenesis in fibroblasts and alveolar epithelial cells, and inflammation induced by ROS and CSE exposure was ameliorated. These results were associated with the inhibition of the NFκB pathway. Collectively, our data indicate that L-carnosine induces anti-inflammatory and anti-fibrotic effects on alveolar epithelial cells against the pathogenesis of IPF.

## 1. Introduction

Idiopathic pulmonary fibrosis (IPF) is a type of chronic lung disease characterized by epithelial cell injury, extracellular matrix (ECM) remodeling, and a progressive decline in lung function [1]. In addition, IPF has a poor prognosis with an estimated median survival of 3–5 years post-diagnosis [1]. The pathogenesis of IPF is induced by several factors such as epithelial cell aging caused by recurrent epithelial injury and interstitial collagen deposition of myofibroblast caused by many risk factors such as cigarette smoke exposure, bacterial or viral infection, and exposure to environmental particles [2,3,4]. The development of IPF is associated with shortened telomeres, proteostatic dysregulation, oxidative stress, mitochondrial dysfunction, epithelial-to-mesenchymal transition, macrophage accumulation, and endoplasmic reticulum stress [2,3]. Two types of anti-fibrotic drugs (pirfenidone and nintedanib) have been shown to decrease disease progression; however, these drugs could not completely treat IPF [5]. Therefore, IPF treatment has remained a challenge.

Reactive oxygen species (ROS) are generated in normal aerobic states as a natural byproduct. ROS is necessary for cell signaling and homeostasis which is associated with host defense, cellular response to injury, and cell differentiation; however, excessive ROS can adversely affect cells. Therefore, ROS balance is important [6,7]. Mitochondria and NADPH oxidase (NOX) are the main sources of ROS, which can indicate IPF pathogenesis [8]. For example, dysfunction of mitochondria was observed in IPF and it can contribute to IPF development by producing mitochondrial ROS. In addition, Nox2 and Nox4 were regarded as an important factor in IPF because of their association with fibroblast differentiation, alveolar epithelial cell death, and mitochondrial dysfunction. Therefore, if the balance of ROS breaks down, then ROS can contribute to the development of IPF [9,10]. Increased ROS through Nox2 or Nox4 can activate p53 and it led to PARP cleavage, DNA fragmentation, Caspase-3 activation, and NFκB activation [11,12,13,14,15]. Nox2 or Nox4 increases in patients, with IPF and exacerbates IPF development through alveolar epithelial cell death, myofibroblast differentiation, and collagen deposition [9]. Furthermore, the deletion of Nox2 or Nox4 in mice is protected from BLM-induced lung fibrosis [16]. P53 activation promotes pulmonary fibrosis by cellular apoptosis, cell aging, oxidative stress, and epithelial mesenchymal transition. P53 is upregulated in patients with IPF, and it is related to poor prognosis [17]. Activated caspase-3, a marker of apoptosis, co-localizes significantly in IPF lungs rather than normal lungs, and it is related to the decrease in lung function [18]. NFκB activation promotes tissue remodeling by myofibroblast differentiation [19]. NFκB activation also induces TGFβ gene transcription [20] and inhibition of NFκB activation protected from BLM-induced lung fibrosis [21]. Furthermore, NFκB is activated in patients with IPF [22].

L-carnosine (Lcar; carnosine) is a dipeptide that consists of β-alanine and L-histidine, which can be found in various tissues such as the skeletal muscle, kidneys, cardiac muscle, and brain. L-carnosine is known as an intracellular pH buffer and it has other physiological functions. L-carnosine has also many anti-aging effects, including stimulus mitochondrial activity, slowed telomere shortening, and reduced toxic metabolite or error protein generation [23]. Furthermore, L-carnosine shows therapeutic effects in many diseases such as neurodegenerative diseases, type II diabetes, acute kidney failure, and colorectal cancer [23,24,25,26]. In particular, L-carnosine has a therapeutic effect on fibrotic diseases, including liver and renal fibrosis [27,28].

In lung fibrosis, L-carnosine suppressed nitrotyrosine and transforming growth factor-β (TGF-β) which showed therapeutic potential for IPF [29]. However, this effect was only examined for 7 days. For bleomycin (BLM)-induced IPF murine model, integral fibrosis occurred 2 weeks after BLM administration [30]. Therefore, it is earlier to show the therapeutic effect of L-carnosine on IPF. In addition, the experiment focused only on the therapeutic effects of L-carnosine. Therefore, we analyzed the therapeutic effects and biological mechanism of L-carnosine on integral fibrosis. The result showed that L-carnosine protected alveolar epithelial cell apoptosis from BLM-induced ROS via ROS regulation.

## 2. Materials and Methods

### 2.1. Animal Studies

C57BL/6 mice (8 weeks/male, 22–25 g) were purchased by Doo Yeol Biotech (Seoul, Republic of Korea). All animal experiments have followed the regulations of the Institutional Animal Care and Use Committee (KW-200309-1, Kangwon National University, Chuncheon-si, Republic of Korea). These animals were randomly divided into four groups (CTL, Control; L-car, L-carnosine; BLM, Bleomycin). The animals were anesthetized with Zoletil (Virbac Corp., Westlake, TX, USA) and intra-tracheal injected with Bleomycin (3 mg/kg, B3972, Tokyo Chemical Industry, Tokyo, Japan) in 50 μL of saline. After 4 h, the mice were injected with oral administration L-carnosine (150 mg/kg, C9625, Sigma-Aldrich, St. Louis, MO, USA) in 200 μL of 1X PBS each day. The body weight of each group was measured every 2 days. After 15 days, lungs were retrieved and measured for weight.

### 2.2. Bronchoalveolar Lavage Fluid (BALF)

After 15 days, mice were sacrificed and BALF was isolated with a 22-gauge intravenous catheter and 1 mL of saline into the trachea. The lungs were lavaged 1 time with 1 mL PBS. BALF was centrifuged (3000 rpm, 10 min, 4 °C) and separated supernatant. Additionally, then, the bronchoalveolar lavage fluid (BAL) cell pellet was collected and resuspended 1 mL 1X PBS. After the total cell number was determined with a hemocytometer, the BAL cells were attached on poly-L-lysine-coated slides (Sigma-Aldrich, St. Louis, MO, USA) using cytospin centrifuge (3000 rpm, 5 min). For immune cell counts, the cells were stained with HEMA3 (Thermo Fisher Scientific, Waltham, MA, USA). In brief, the slide was stained in HEMA 3 Fixative for 5 min. Additionally, washed with distilled water, dipped in HEMA 3 Solution I for 5 min, and washed again with distilled water. Finally, the slide was dipped in HEMA 3 Solution II for 5 s and washed with distilled water. All stained slides were evaluated by microscopy (magnification, ×200). The cells were identified by standard morphology and staining characteristics.

### 2.3. Histological Assessments

The mouse lungs were isolated and fixed using 4% paraformaldehyde overnight. Additionally, then, the lungs were embedded in paraffin and cut into 5 μm sections. For histological assessments, the sections were stained with hematoxylin and eosin (H&E) and Masson’s trichrome (American MasterTech, Lodi, CA, USA). Staining was prepared according to the manufacturer’s protocol. All stained slides were filmed by OCUS40 Microscope Scanner (Grundium, Tampere, Finland) under the same conditions and pictured using SlideViewer (3DHISTECH, Budapest, Hungary).

### 2.4. Ashcroft Score

Morphological changes in lung sections were graded according to Ashcroft score [31]. All lung sections were assessed using a system of 0–8 scores. For assessments, the lung sections consisted of distal and proximal regions. After each section, the assessment averaged all the results.

### 2.5. Quantitative Real-Time Reverse Transcription-Polymerase Chain Reaction (qRT-PCR)

The RNA was extracted from tissues by using Trizol Isolation Reagent (#79306; Qiagen, NRW, Düsseldorf, Germany) according to the manufacturer’s protocol. Additionally, then, the RNA was reverse transcripted into cDNA using Revers Transcription-premix (#EBT-1514; Elpis Biotech, Daejeon, Republic of Korea). The cDNAs were used for qRT-PCR analysis with primer sets in Table 1 and Table 2, and SYBR Green (#RT501; Enzynomics, Daejeon, Republic of Korea), according to the manufacturer’s protocol. Finally, the gene expression of cDNA each group used the StepOnePlus Real-Time PCR systems (Applied Biosystems, Austin, TX, USA). Gene expression was normalized to the Ct values of GAPDH in each sample and was calculated by the 2^−ΔΔCt^ methods.

### 2.6. Western Blot Analysis

The proteins from mouse whole-lung homogenates and whole-cell lysates were made by RIPA lysis buffer. The protein concentration was measured using a Bicinchoninic (BCA) protein assay kit (#23225, Thermo Fisher Scientific, Waltham, MA, USA). Additionally, then, the 20 μg of protein was mixed with a 5X SDS-PAGE sample buffer. The proteins were separated into 6–15% SDS-polyacrylamide gel using electrophoresis. The separated proteins were transferred with nitrocellulose transfer membranes (0.45 μm; #162-0115; Bio-Rad, Hercules, CA, USA). For blocking, the membranes were incubated with 5% skim milk for 1 h. Additionally, then, probed with 1:2000–1:10,000 diluted antibodies: anti-TGFβ1 (sc-130348), anti-Fibronectin (sc-9068), anti-p53 (sc-6243), anti-NFκB (sc-8008), anti-IκBα (sc-371) (Santa Cruz Biotechnology); anti-β-actin (#3700), anti-ERK (#4695), anti-pERK (#4370), anti-JNK (#9252), anti-pJNK (#4668), anti-p38 (#9212), anti-pp38 (#4511), and anti-pNFκB (#3033) (Cell Signaling Technology); anti-α-SMA (ab5694), anti-Nox2 (ab80508), and anti-Nox4 (ab133303) (Abcam, Cambridge, MA, USA) and anti-Caspase-3 (NB100-56708) (Novus biologicals) to detect the respective proteins for 24 h at 4 °C. After being washed 3 times using a TBST buffer (TBS with 0.05% Tween 20), the membranes were incubated with polyclonal anti-rabbit/mouse or goat HRP-conjugated secondary antibodies (NCI1460KR, 31430, 31400; Thermo Fisher Scientific, Waltham, MA, USA) for 1 h at room temperature. After 3 times washing in TBST buffer, the membranes were detected using ECL substrate (#170-5061, BIO-RAD, CA, USA). Protein band expression was measured by ImageJ (National Institutes of Health, Bethesda, MD, USA) analysis and expressed as a fold change relative to β-actin.

### 2.7. Hydroxyproline Assay

For measurement of hydroxyproline in lung tissue, it was performed using hydroxyproline colorimetric assay kit (ab222941, Abcam). For sample preparation, the lung tissues were reacted with 10 N NaOH for 1 h. Additionally, then, it was neutralized with 10 N HCl. Afterward, we followed the manufacturer’s protocol. Briefly, oxidation mixture was added to supernatant and reacted for 20 min. The developer and DAMB were added per well, and reacted at 65 °C for 45 min. Finally, it was measured at 560 nm by a microplate reader (BioTek, Winooski, VT, USA).

### 2.8. Immunofluorescence Staining

For measurement of epithelial cell marker expression in the lung, it was stained with anti-EpCAM (sc-66020, Santa Cruz Biotechnology). The lung-embedded paraffin was sliced into 4 μm sections, and the paraffin was dissolved. It was boiled with Antigen Retrieval solution (0.1 M Sodium Citrate, pH 6) at 121 °C for 1 min. After washed, it reacted with peroxidase buffer (Peroxidase, Methanol). Additionally, then, it was blocked with 10% normal goat serum for 1 h at room temperature and incubated with antibody for overnight. A secondary antibody (anti-mouse IgG, Alexa Fluor 488 conjugate, #A32731, Invitrogen) was reacted for 1 h, and mounted by Fluoroshield with DAPI (#6057, Sigma-Aldrich, St. Louis, MO, USA). Slides were observed by microscope (EVOS M5000; Thermo Fisher Scientific, Waltham, MA, USA) under the same conditions. Relative intensity was measured by ImageJ (National Institutes of Health, Bethesda, MD, USA)

### 2.9. Superoxide Anion Scavenges Activity (SOD Activity)

The superoxide anion scavenging activity was measured by a commercial SOD determination kit (19160 SOD Determination Kit, Sigma-Aldrich, St. Louis, MO, USA), according to the manufacturer’s protocol. The inhibition rate of O2 reduction by SOD was measured using the production of a water-soluble tetrazolium salt, WST-1 [2-(4-iodophenyl)-3-(4-nitrophenyl)-5-(2,4-disulfophenyl)-2H-tetrazolium, monosodium salt] formazan dye in the presence of superoxide anions (O^2−^). The results indicate the inhibition activity of SOD.

### 2.10. Preparation of Cigarette Smoke Extract (CSE)

Research-grade cigarettes (3R4F) were obtained from the Kentucky Tobacco Research Development Center (The Tobacco Research Institute, University of Kentucky, Lexington, KY, USA). The composition of 3R4F research grade cigarettes was: 11.0 mg total particulate matter, 9.4 mg tar, and 0.73 mg nicotine each cigarette. The 3R4F cigarette was fixed in inhalation entrance of a peristaltic vacuum pump (im-pingers) (WP6122050, Merck, Darmstadt, Germany). After igniting cigarettes, the pump was constantly inhaled by vigorous bubbling, at the same time, smoke was blended with 10 mL serum-free DMEM/F-12 media for 1–2 min. Additionally, then, the media containing smoke was filtered through a 0.22 μm filter (16534 K, Sartorius Minisarts, Goettingen, Germany) to remove large particles and was regarded as a 10% CSE solution. Cigarette smoke extract was freshly prepared for each experiment and diluted with culture media supplemented with 1% FBS immediately before use.

### 2.11. Cell Culture

Alveolar epithelial type II cell line was used for this study along with the mouse alveolar epithelial cell line (MLE-12) and obtained from the American Type Culture Collection (CRL-2110, Manassas, VA, USA). MLE-12 cells were cultured in DMEM/F-12 media with 1% penicillin-streptomycin and 2% FBS. In addition, DMEM/F-12 media for MLE-12 cells was supplemented with 0.005 mg/mL insulin, 30 μM sodium selenite, 10 nM hydrocortisone, and 10 nM β-estradiol at 37 °C and 5% CO_2_.

In order to induce epithelial mesenchymal transition, MLE-12 cells were treated with recombinant mouse 10 ng/mL TGFβ (#Cyt-858; Prospec, Ness-Ziona, Israel) for 72 h. For the inhibitory effect of L-carnosine, MLE-12 cells were co-treated with 30 mM of L-carnosine and 10 ng/mL TGFβ for 72 h. The human fibroblast cell line, MRC-5 cells, were obtained from the Korean Cell Line Bank (Seoul, Republic of Korea). It was incubated in DMEM/High glucose media with 1% penicillin-streptomycin and 10% FBS at 37 °C and 5% CO_2_. To induce myofibroblast differentiation, MRC-5 cells were treated with recombinant human 5 ng/mL TGFβ (#Cyt-716; Prospec, Ness-Ziona, Israel) for 48 h. For the inhibitory effect of L-carnosine, MLE-12 cells were co-treated with 10 mM of L-carnosine and 5 ng/mL TGFβ for 48 h.

### 2.12. MTT Assay

MLE-12 cells were exposed to 0.5% of CSE and co-treated with L-carnosine in the range of 5 and 30 mM in a 24-well plate for 24 h. After 24 h, the culture media were removed and wash with 1X PBS. The MLE-12 cells were next treated with 20 μL/mL of 3-(4,5-dimethylthiazol-2-yl)-2,5-diphenyltetrazolium bromide (MTT) reagents for 3 h. After removing the MTT reagent, the cells were added to dimethylsulfoxide (DMSO) to dissolve formazan crystals. The absorbance was read at 540 nm on a microplate reader (BioTek, VT, USA). The absorbance was measured at 540 nm using a microplate reader, and cell viability was calculated using the following equation: cell viability (%) = (A_treatment_ − A_blank_)/(A_control_ − A_blank_) × 100%, A = absorbance, OD value.

### 2.13. Immunostaining

The MLE-12 cells were treated with L-carnosine (30 mM) and CSE (0.25%) for 24 h at 37 °C and 5% CO_2_ in the coating cover slide. After washed, the MLE-12 cells were fixed by 4% paraformaldehyde for 10 min and were treated with 0.2% Triton X-100 for 10 min for permeabilization. Additionally, then, the slides were blocked with 10% normal goat serum for 1 h at room temperature. After removing normal goat serum, anti-Ki67 (#9129, Cell Signaling Technology) antibody was diluted with 1% normal goat serum overnight. A secondary antibody was used to anti-rabbit IgG (Alexa Fluor 488 conjugate, #4412, Cell signaling) for 1 h. Finally, slides were mounted by Fluoroshield with DAPI (#6057, Sigma-Aldrich, St. Louis, MO, USA) and observed under the confocal microscope (K1-Fluo; Nanoscope Systems, Daejon, Republic of Korea) under the same conditions.

### 2.14. Detection of Intracellular Reactive Oxygen Species (ROS)

MLE-12 cells cultured in 6-well plates were incubated in a 10 μM solution of 2′7′-dichlorodihydrofluorescein diacetate (DCFDA, Sigma-Aldrich, St. Louis, MO, USA, D6883) at 37 °C for 30 min (Thermo Fisher Scientific, Waltham, MA, USA), and subsequently exposed to 0.5% of CSE and/or 30 mM of L-carnosine for 24 h. After 30 min of exposure, ROS production for 20,000 ungated events were collected and quantified by measuring the relative fluorescent intensity (RFI) of DCFDA on an Accuri-C6 flow cytometry (BD Biosciences, Franklin Lakes, NJ, USA). Briefly, the fluorescence expression of DCF-DA in FL1-A channel was transformed to a percentage using FlowJo v8 software.

### 2.15. Statistical Analysis

All statistical analyses were performed with GraphPad Prism 9.0 (GraphPad Software, San Diego, CA, USA). All statistics were used by One-way analysis of variance (ANOVA) for Bonferroni post hoc test. A value of * *p* < 0.05, ** *p* < 0.01, and *** *p* < 0.001 was compared with each indicated group for all pairs.

## 3. Results

### 3.1. L-carnosine Rescued Pulmonary Edema in Bleomycin-Induced Fibrosis via Immune Cell Infiltration

**Figure 1:**
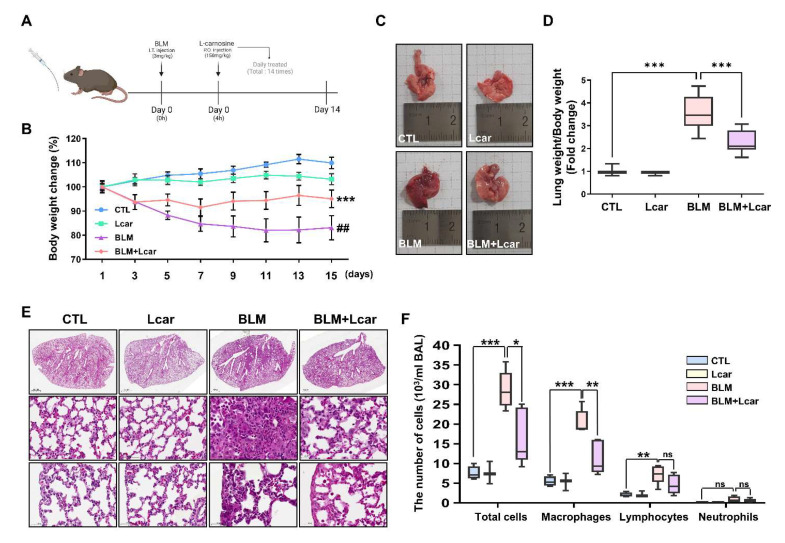
L-carnosine decreased pulmonary edema in bleomycin-induced fibrosis via inhibition of immune cell infiltration. For the IPF mouse model, C57BL/6 mice were injected intratracheally with bleomycin (3 mg/kg). After 4 h, L-carnosine was injected with oral administration each day. After 15 days, mice were sacrificed and lungs were harvested. (**A**) Timetable of experiment schedule. (**B**) Body weight change of each group for 15 days (*n* = 5/group). *** *p* < 0.001 compared with the control group by one-way ANOVA. ## *p* < 0.01 compared with the BLM group by one-way ANOVA (**C**) Representative images of the harvested lung at each group. (**D**) Lung weight was divided by body weight (*n* = 7/group). (**E**) The lung tissue was stained using H&E staining. It was pictured various regions including whole lung (**top**), proximal (**middle**), and distal (**bottom**). (**F**) Counting each cell type in BALF using cytospin and Giemsa staining. BAL cells were obtained from bronchoalveolar lavage fluid. (*n* = 4,3,5,5/group) A value of * *p* < 0.05, ** *p* < 0.01, and *** *p* < 0.001 was compared with each indicated group by one-way ANOVA. I.T, Intratracheal; P.O, Per oral; CTL, Control; L-car, L-carnosine; BLM, Bleomycin; Scale bar: 1000 μm (**top**) or 50 μm (**middle**, **bottom**).

In determining whether L-carnosine inhibits inflammation, bleomycin (BLM) was intratracheally administered mice (Figure 1A). The mice treated with BLM and L-carnosine (Lcar) showed a decrease in body weight at 3 days, whereas the other mice maintained their bodyweight (Figure 1B). The gross and histological changes of BLM-exposed lungs exhibited infiltrated cells into the alveoli space and thickened alveolar epithelium compared with the CTL group. By contrast, the BLM + Lcar group ameliorated infiltrated cells and thickened the alveolar epithelium based on H&E staining analysis (Figure 1C,E). In addition, BLM-exposed mice significantly increased the mean lung-to-body-weight ratio, whereas mice treated with BLM and Lcar decreased the lung-to-body-weight ratio (Figure 1D). Differential cell counts in bronchoalveolar lavage fluid were determined 14 days after the BLM instillation. The infiltration of macrophages and lymphocytes significantly increased in BLM-exposed mice, whereas it was inhibited in mice treated with BLM and Lcar (Figure 1F). These findings indicate that Lcar ameliorates pulmonary edema via the inhibition of macrophage and lymphocyte infiltration.

### 3.2. L-carnosine Treatment Attenuates Collagen Production in Mice Treated with Bleomycin

**Figure 2:**
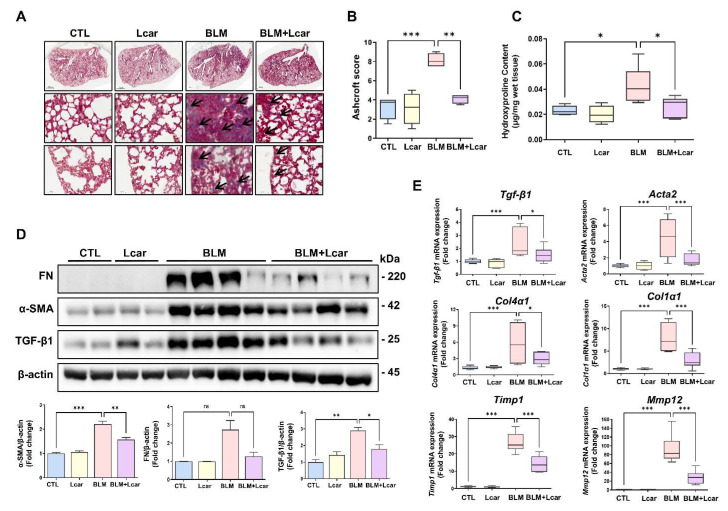
L-carnosine downregulated collagen deposition in bleomycin-induced fibrosis. It was pictured various regions including whole lung (**top**), proximal (**middle**), and distal (**bottom**). (**A**) The lung tissue was stained using M/T staining. Pathological lesions were indicated with black arrows (**B**) Ashcroft score of each group. (**C**) Hydroxyproline contents in tissue of each group (*n* = 4/group). (**D**) Protein expression of Fibronectin, α-SMA, and TGF-β was measured by Western blot and relative density ratio. (**E**) mRNA expression of fibrosis-related genes was measured by real-time PCR (*n* = 4/group). A value of * *p* < 0.05, ** *p* < 0.01, and *** *p* < 0.001 was compared with each indicated group by one-way ANOVA. CTL, Control; L-car, L-carnosine; BLM, Bleomycin; FN, Fibronectin. Scale bar: 1000 μm (**top**) or 50 μm (**middle**,**bottom**).

In determining whether Lcar treatment ameliorates lung fibrosis induced by BLM, the lung sections of each group were stained with Masson’s trichrome. BLM-exposed mice showed that the area of collagen fiber was remarkably increased and distributed in areas of focal consolidation. In addition, the structure of the alveolus was damaged. Focal consolidation and alveolar atrophy also collapsed and thickened. By contrast, in the BLM + Lcar group, the Ashcroft score including collagen deposition, significantly decreased (Figure 2A,B). In assessing collagen contents in tissues, we performed a hydroxyproline assay. Consequently, such contents were increased in BLM groups, but Lcar treatment led to a significant reduction (Figure 2C). Moreover, BLM-exposed mice treated with Lcar showed a significant decrease in α-SMA, and TGF-β1 protein levels (Figure 2D). Furthermore, mRNA levels of *Tgf-β1*, *Acta2*, *Col4α1*, *Col1α1*, *Timp1*, and *Mmp12* increased, but they were significantly inhibited by Lcar treatment (Figure 2E). These results indicate that Lcar treatment can attenuate BLM-induced production of ECM proteins.

### 3.3. L-carnosine Treatment Protects Alveolar Injury via Nox4-p53 and Caspase-3 Mediated Apoptosis

**Figure 3:**
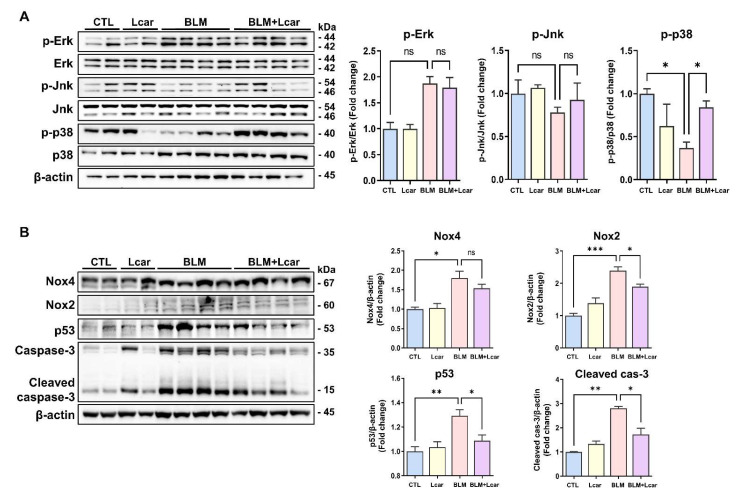
L-carnosine treatment protected alveolar injury via Nox2, Nox4-p53 and Caspase-3 mediated apoptosis. (**A**) Each protein was measured by Western blot. Relative density ratio graph of p-Erk, p-Jnk, and p-p38. All proteins were divided by each total form protein. (**B**) Proteins were measured by Western blot. Relative density ratio graph of Nox2, Nox4, p53, and Cleaved caspase-3. All proteins were divided by β-actin. A value of * *p* < 0.05, ** *p* < 0.01, and *** *p* < 0.001 was compared with each indicated group by one-way ANOVA. CTL, Control; L-car, L-carnosine; BLM, Bleomycin; cas-3, caspase-3.

Given the anti-inflammatory and anti-fibrotic effect of Lcar on pulmonary fibrosis, we aimed to define the molecular mechanism underlying the role of Lcar in BLM-induced fibrosis. Phosphorylation of Erk protein expression was increased in the BLM group; however, Lcar treatment did not change the elevated Erk activation. The protein level of Jnk was not altered in response to BLM or Lcar treatment. By contrast, BLM significantly decreased the phosphorylated p38 protein level, whereas Lcar treatment led to an increase in BLM-exposed lungs of mice (Figure 3A). In previous studies, alveolar epithelial cells were damaged by ROS imbalance in mice with BLM-induced fibrosis [9]. In addition, Nox2, Nox4, and p53 proteins contribute to ROS-related injury [32]. Therefore, we determined Nox2, Nox4 and p53 protein levels, which are related to the p38 protein-associated mechanism. The Nox2 and Nox4 protein expression, as the major sources of ROS production, and p53 protein expression were significantly increased in the BLM group compared with the CTL group. Moreover, the cleaved caspase-3 protein level was significantly increased in the BLM group (Figure 3B). Lcar treatment downregulated Nox2, Nox4, and p53 protein levels and decreased active caspase-3 in BLM-exposed mice (Figure 3B). These data indicate that Lcar treatment downregulated Nox2 and Nox4 expression levels, which restored of ROS, thereby inhibiting apoptosis via decreasing caspased-3 and p53 protein level.

**Figure 4:**
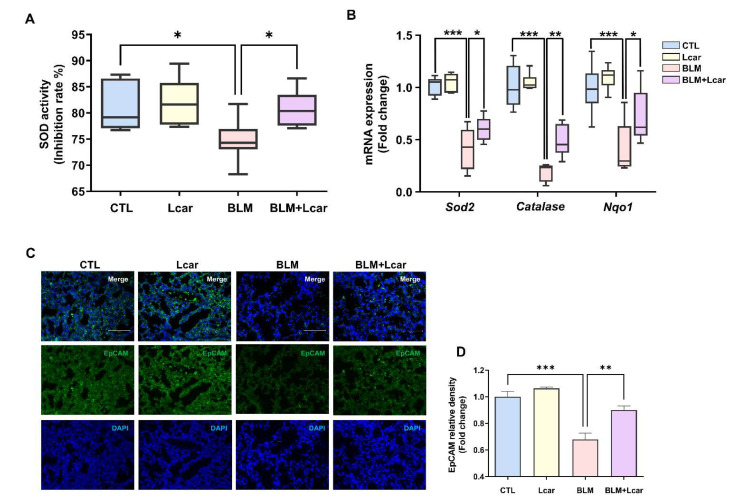
L-carnosine improved ROS-induced anti-oxidant activity. (**A**) SOD activity was measured by the SOD determination kit (*n* = 4/group). (**B**) mRNA expression of antioxidant-related genes was measured by real-time PCR (*n* = 4/group). (**C**) Representative images of immunofluorescence staining of lung tissues using EpCAM antibody. (**D**) Relative density expression graph as fold change relative to control group (*n* = 4/group). A value of * *p* < 0.05, ** *p* < 0.01, and *** *p* < 0.001 was compared with each indicated group by one-way ANOVA. CTL, Control; L-car, L-carnosine; BLM, Bleomycin; Scale bar: 30 μm.

In evaluating the anti-oxidant effect of Lcar, the superoxide anion scavenging activity and mRNA level of anti-oxidant genes were determined. Lcar treatment significantly restored superoxide anion scavenging activity in BLM exposed lungs of mice (Figure 4A). Moreover, Sod2, Catalase, and Nqo1 were significantly decreased in BLM-exposed mice, whereas Lcar treatment elevated mRNA levels of Sod2, Catalase, and Nqo1 (Figure 4B). These findings indicate that Lcar treatment diminishes oxidative stress via upregulating the anti-oxidant activity.

During IPF pathogenesis, epithelial cell elimination can exacerbate disease development. As shown in Figure 3B, Lcar treatment prevented apoptosis and ameliorated IPF. However, whether Lcar rescued epithelial cells from BLM-induced oxidative injury remains unclear. With regard to the epithelial cell injury, we measured epithelial cell marker expression using immunofluorescence staining. EpCAM, markers of various epithelial cells, was decreased by BLM challenge (Figure 4C,D). By contrast, Lcar treatment enhanced EpCAM expression. Collectively, we focused on epithelial cells, and further study was conducted on injury and fibrogenesis in epithelial cells.

### 3.4. L-carnosine Downregulated Fibrogenesis in Fibroblasts and Lung Epithelial Cells

We demonstrated the anti-fibrotic and anti-inflammatory effects on experimental IPF mouse model. In IPF, fibrosis results from fibroblast differentiation into myofibroblast and epithelial-mesenchymal transition [1,2,3]. Therefore, we determined the anti-fibrotic efficacy on the cell level for excluding tissue environment.

**Figure 5:**
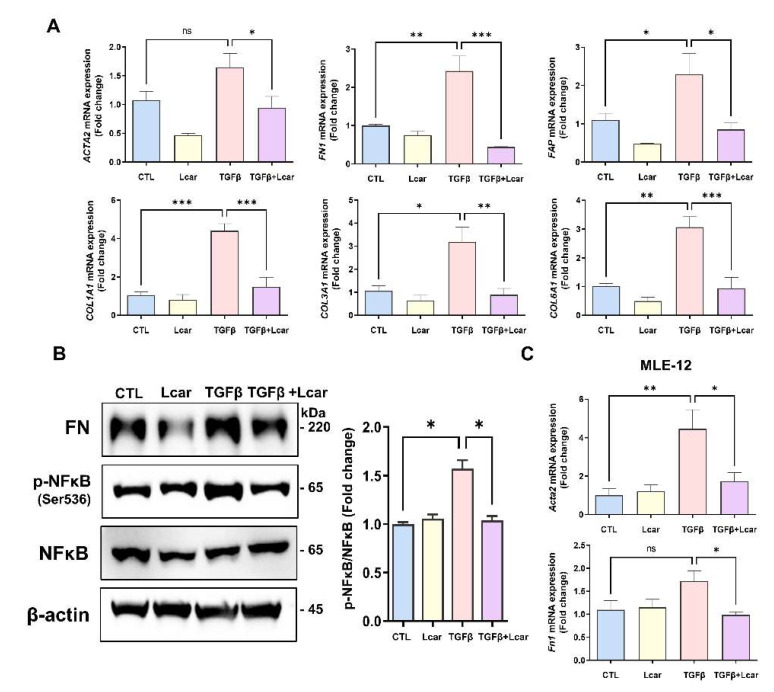
L-carnosine downregulated fibrogenesis in fibroblasts and lung epithelial cells. Human lung fibroblast (MRC-5) and mouse lung epithelial cell (MLE-12) were stimulated with human recombinant TGFβ and mouse recombinant TGFβ for 48 h and 72 h, respectively. L-carnosine was treated MRC-5 (10 mM) and MLE-12 (30 mM) at the same time. (**A**) mRNA expression of fibrosis-related genes including ACTA2, FN1, FAP, COL1A1, COL3A1, and COL6A1 in MRC-5 was measured by real-time qPCR. (**B**) Protein expression of FN and NFκB was measured by Western blot analysis, and relative intensity ration graph was presented. (**C**) mRNA expression of Acta2 and Fn1 in MLE-12 cells were measured by real-time PCR. A value of * *p* < 0.05, ** *p* < 0.01, and *** *p* < 0.001 was compared with each indicated group by one-way ANOVA. CTL, Control; L-car, L-carnosine; FAP, Fibroblast activation protein.

MRC-5 (human fibroblast) and MLE-12 (mouse lung epithelial cell) were prepared. Afterward, they were stimulated with human recombinant TGFβ and mouse recombinant TGFβ. Consequently, the level of fibrosis-related genes and proteins increased during TGFβ stimulation followed by NFκB phosphorylation induction in MRC-5 cells (Figure 5A,B). After Lcar treatment, fibrosis-related genes including ACTA2, FN1, FAP, COL1A1, COL3A1, and COL6A1 were significantly decreased compared with the TGFβ-stimulated group. Furthermore, the phosphorylation of NFκB at Ser536 was significantly decreased (Figure 5B). In addition, MLE-12 cells induced fibrogenesis because of TGFβ stimulation (Figure 5C). Lcar also affected MLE-12 cells by reducing fibrosis-related genes, including Acta2 and Fn1.

### 3.5. L-carnosine Rescued Alveolar Epithelial Cells in Cigarette Smoke Extract Exposure by Inhibiting ROS Production through Diminishing NFκB Signaling Pathway

We confirmed the anti-fibrotic effect of Lcar on fibroblast and lung epithelial cells. Except for fibrogenesis, alveolar epithelial cell apoptosis is a prominent feature of pulmonary fibrosis. Therefore, we analyzed the anti-inflammatory effect of Lcar on the IPF experimental mouse model, through CSE stimulation.

**Figure 6:**
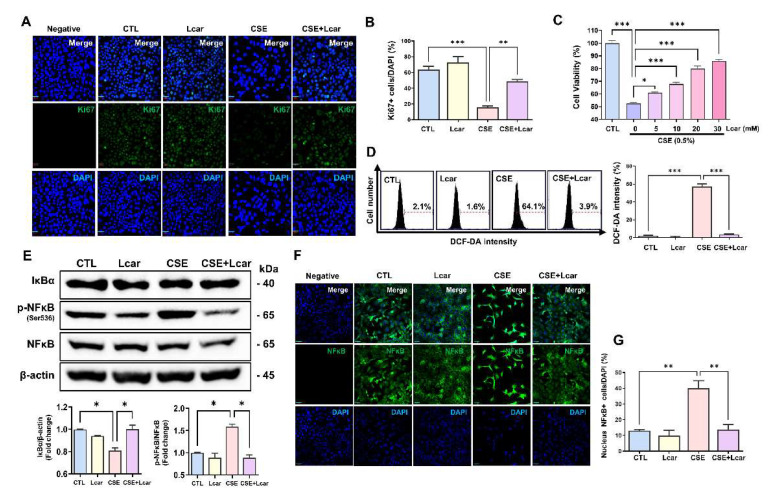
L-carnosine rescued alveolar epithelial cells in cigarette smoke extract exposure by inhibiting ROS production through diminishing NFκB signaling pathway. Mouse lung epithelial cell (MLE12) was exposed to 0.25% cigarette smoke extract for 24 h. At the same time, L-carnosine (5 mM~30 mM) was co-treated. (**A**) Representative images of immunocytochemistry assay. Ki67 was labeled by GFP. (**B**) Relative fluorescence intensity ratio graph of Ki67 (*n* = 3/group). (**C**) Cell viability was measured by MTT assay (*n* = 4/group). (**D**) Representative images of DCF-DA assay. ROS generation was measured by DCF-DA assay, and relative fluorescence intensity ratio graph of DCF-DA (*n* = 3/group). X axis means FL-A and y axis means count of cells. (**E**) Protein expression of NFκB and IκBα was measured by Western blot. Relative density ratio graph of NFκB and IκBα. IκBα was divided by β-actin and p-NFκB was divided by NFκB (*n* = 3/group). (**F**) Representative images of immunocytochemistry assay. NFκB was labeled by GFP. (**G**) Nucleus NFκB+ cell was divided by DAPI (*n* = 3/group). A value of * *p* < 0.05, ** *p* < 0.01, and *** *p* < 0.001 was compared with each indicated group by one-way ANOVA. CTL, Control; L-car, L-carnosine; CSE, Cigarette smoke extract. Scale bar: 30 μm.

Cigarette smoke is a major risk factor in IPF, which is widely used in IPF-related experiments such as myofibroblast differentiation, alveolar epithelial cell senescence, and mouse fibrosis model [33,34,35]. MLE-12 cells were exposed CSE for 24 h. In evaluating cellular proliferation, we performed Ki67 immunocytochemistry using MLE-12 cells treated with CSE or Lcar. Ki67 expression was significantly decreased in response to CSE, however, L-carnosine treatment induced higher Ki67 expression in MLE-12 cells treated with CSE rather than single CSE treatment (Figure 6A,B). In cell viability assay, 0.5% CSE treatment significantly inhibited the cell viability of MLE-12 cells, whereas Lcar treatment increased the cell viability in a dose-dependent manner (Figure 6C).

We confirmed that Lcar treatment enhanced anti-oxidant genes thereby ameliorating BLM-induced alveolar injury in mice. In determining whether Lcar treatment regulates ROS production in alveolar epithelial cells, we measured ROS generation in CSE-exposed MLE-12 cells using the DCF-DA assay. As shown in Figure 6D, CSE increased DCF-DA fluorescence, whereas CSE treated with Lcar significantly decreased the DCF-DA intensity. We also determined whether Lcar-mediated ROS inhibition is associated with the NFκB pathway. In Western blot analysis, CSE increased the phosphorylation of NFκB at Ser536 and degradation of IκBα proteins. Lcar treatment blocked IκBα degradation and NFκB phosphorylation at Ser536 in CSE-treated MLE-12 cells (Figure 6E). In addition, Lcar treatment significantly inhibited CSE-induced nuclear translocation of NFκB in immunocytochemistry (Figure 6F,G). These data indicate the inhibitory effect exerted by Lcar on ROS production and activation of the NFκB pathway.

## 4. Discussion

In this study, we demonstrated the anti-fibrotic and anti-inflammatory effects of Lcar and its therapeutic pathways in mice and alveolar epithelial cells. In particular, this study has shown the effect of carnosine in mice with BLM-induced lung injury [29]. However, it is limited with the anti-inflammatory effect of carnosine at 7 days, which may serve as an add-on therapy of fibrotic disorder. Therefore, we determined the anti-inflammatory effect via the inhibition of inflammatory cell infiltration and further investigated anti-fibrotic role in BLM-induced pulmonary fibrosis. Furthermore, we found that Lcar treatment increased cell proliferation, viability, and inhibition of ROS production against CSE exposure.

The major feature of IPF is the accumulation of macrophages. Alveolar macrophages are key components of the innate immune system, which exhibit extensive plasticity. They can polarize M1 or M2 states under certain molecular mediators under various micro-environments. M1 macrophages are classically activated by LPS or IFN-γ and they contribute to host defense by producing reactive nitric oxide and inflammatory cytokines and chemokines such as IL-1β, IL-6, IL-12, CCL2, and TNF-α [36]. M2 macrophages are alternatively activated by IL-4, IL-10, and IL-13 and they maintain tissue repair by producing anti-inflammatory cytokines [37]. During fibrosis, M2 macrophages infiltrate into the injured area to regulate fibrogenesis [38,39]. Moreover, M1 macrophages slowly switch into M2 phenotype leading to fibrosis by secreting various mediators [40]. Therefore, exudate macrophages and Ly-6C^high^ monocytes are recruited, which contribute to IPF pathogenesis [41,42]. In our data, in BLM challenge, Lcar treatment reduced the infiltrated macrophages, neutrophils, and lymphocytes in the lung tissue. This finding indicates that carnosine modulates macrophage-induced nitric oxide and alters M1/M2 macrophage polarization [43,44]. Accordingly, our data suggest that Lcar treatment decreases NO production by altering M1/M2 macrophages with BLM.

The balance between the production and scavenging of ROS maintains homeostasis and it is involved in cellular differentiation and innate immunity. In IPF, the ROS scavenging capacity declines, which indicates IPF pathogenesis, thereby activating profibrotic cytokine TGF-β. In particular, the ROS production of Nox2 or Nox4 can indicate alveolar epithelial cell injury. Moreover, Nox4 or Nox2-deficient mice were protected from BLM-induced lung fibrosis [16]. In BLM-exposed mice, Nox2 and Nox4 protein expression levels increased, thereby decreasing the SOD activity and anti-oxidant gene expression level, including *Sod2*, *Catalase*, and *Nqo1*. In line with previous studies, our data showed that the level of Nox2 and Nox4 increased with the increase of p53 and cleaved Caspase-3 protein expression level in BLM-exposed lungs of mice. In addition, we found that continuous Lcar treatment reduced Nox2 and Nox4 expression level, thereby decreasing p53 and cleaved Caspase-3 protein expression levels.

p38 MAPK protein regulates cell cycle progression via the modulation of p53 protein [45]. In IPF lungs, p53 protein regulates cell cycle, DNA damage, and apoptosis [17]. In addition, p38 protein-deleted fibroblasts showed a higher density than normal fibroblasts via contact inhibition [46]. Therefore, a decrease in p38 protein is associated with the accumulation of fibroblasts in the pathogenesis of IPF. In addition, p38 knockout fibroblast contained considerable ROS compared with normal cells, and it was related to ERK phosphorylation induction [47,48]. Moreover, cleaved Caspase-3 significantly increased with the increase of p53 protein of BLM-exposed lungs. Therefore, the activation of p53 protein induces nuclear DNA fragmentation, PARP cleavage, and Caspase-3 activation in cancer cells [11]. When IPF mice were treated with Lcar, p53 and Caspase-3 protein activation decreased. These data indicate that Lcar treatment has a protective effect via ROS regulation against BLM-induced apoptosis. In this study, MLE-12 cells were exposed to CSE which is a potent cause of IPF, ROS generation, inflammation, and lung damage via NFκB signal [4,49,50]. Our data demonstrated that Lcar treatment improved the proliferation and viability of MLE-12 cells via inhibition of ROS production. As apoptotic epithelial cells indicate IPF exacerbation [49,50], the protective effect of alveolar epithelial cells play a pivotal role in the progression of pulmonary fibrosis.

In general, fibroblast has been regarded as a major cell type of various fibrosis diseases. In IPF, fibroblast senescence is critical for pathogenesis. Isolated lung fibroblasts from IPF patients exhibited senescence phenotypes such as expression of P53, P21, P15, and senescence-associated secretory phenotypes (SASP) compared with age-matched controls [51]. In addition, cellular senescence can influence tissue integrity by disrupting tissue structure and tissue microenvironment. Furthermore, diminish of senescent cells led to amelioration of aging-related disorders in mouse models [52]. Therefore, regulation of senescence is important. In Figure 5A,B, we demonstrated the anti-fibrotic effect of Lcar in fibroblast. Except for this, L-carnosine can ameliorate fibroblast senescence and modulate various pathways such as TCA cycle, oxidative phosphorylation, apoptosis, and ECM reorganization [53]. Furthermore, L-carnosine treatment led to p53 protein reduction in mice (Figure 5B).

The NFκB pathway is involved in airway remodeling, and mesenchymal transition via epigenetic reprograming of fibrogenic genes [54]. Moreover, its activated pathway induces myofibroblast differentiation, thereby exacerbating IPF [55]. We have shown that Lcar treatment induced a protective effect against CSE exposure via the in-activation of the NFκB pathway in MLE-12 cells. These findings indicate that Lcar induced ROS inhibition is associated with protection from alveolar epithelium loss. Furthermore, fibroblast differentiation into myofibroblast decreased with the decrease of fibrosis-related gene expression level. Collectively, the NFκB pathway is an important pathway of inflammation and fibrogenesis in IPF.

In general, BLM has been used to induce the IPF animal model after it was recognized as a drug that can occur pulmonary fibrosis in some patients. It induces inflammatory response that lasts approximately 7–10 days and is transformed into fibrotic response by inducing TGF-β [56]. Furthermore, the BLM-induced IPF animal model showed histological hallmarks of IPF such as the obliteration of the alveolar space, mural incorporation of collagen, and intra-alveolar buds [57]. Although it reflects some IPF features, it has significant limitations in understanding the progression of IPF in humans. The slow and irreversible progression of IPF in patients is not reflected in the BLM model [58]. Nevertheless, BLM is a certain agent to induce the IPF mouse model in pre-clinical trials. Although BLM has various limitations, we used BLM for mimicking the IPF mouse model in accordance with the aforementioned previous studies.

In conclusion, Lcar prevented alveolar epithelial cells from ROS imbalance caused by Nox4 and Nox2 expression and further improved proliferation, viability, and ROS generation in response to CSE exposure. Considering that increased ROS is a crucial problem of IPF development in human [59], Lcar may attenuate IPF development in human by inhibiting ROS efficacy. Furthermore, increased target proteins including NOX2, NOX4, P53, Caspase-3, and NFκB, can indicate human IPF development [9,17,18,22]. Lcar had an anti-fibrotic effect on fibroblasts and alveolar epithelial cells. Therefore, our findings indicate the potential therapeutic application of Lcar in alveolar epithelial injury and pulmonary fibrosis.

## Figures and Tables

**Table 1:** Mouse primer for used qRT-PCR.

Target Gene	Forward	Reverse
*Tgf-β1*	5′-AGCAGTGAGCGCTGAACTG-3′	5′-GCAGTGGCTGAACCAAGGA-3′
*Acta2*	5′-AGGGACAGCACAGCCTGAAT-3′	5′-CGGGAGAAAATGACCCAGAT-3′
*Col1α1*	5′-CCCGGTGACACACAAAGACA-3′	5′-GACCGTTCTATTCCTCAGTGCA-3′
*Col4α1*	5′-GATGGCGGTACACAGTCAGA-3′	5′-ATCCACAGTGAGGACCAACC-3′
*Timp1*	5′-GCAAAGAGCTTTCTCAAAGACC-3′	5′-GGGATAGATAAACAGGGAAACACT-3′
*Mmp12*	5′-TGATGCAGCTGTCTTTGACC-3′	5′-GTGGAAATCAGCTTGGGGTA-3′
*Sod2*	5′-GGCCAAGGGAGATGTTACAA-3′	5′-GCTTGATAGCCTCCAGCAAC-3′
*Catalase*	5′-CCTCGTTCAGGATGTGGTTT-3′	5′-AGGAATCCGCTCTCTGTCAA-3′
*Nqo1*	5′-TCGGGCTAGTCCCAGTTAGA-3′	5′-AAGTTAGTCCCTCGGCCATT-3′
*Gapdh*	5′-CGCTCCTGGAAGATGGTGAT-3′	5′-GGCAAATTCAACGGCACAGT-3′

**Table 2:** Human primer for used qRT-PCR.

Target Gene	Forward	Reverse
*ACTA2*	5′-TTCAATGTCCCAGCCATGTA-3′	5′-GCAAGGCATAGCCCTCATAG-3′
*COL1A1*	5′-GGGATTCCCTGGACCTAAAG-3′	5′-CTCCAGCCTCTCCATCTTTG-3′
*COL3A1*	5′-AGGGGAGCTGGCTACTTCTC-3′	5′-CCTCCTTCAACAGCTTCCTG-3′
*COL6A1*	5′-ACCTACACCGACTGCGCTAT-3′	5′-TCGGTCACCACAATCAGGTA-3′
*FAP*	5′-GGCTCACGTGGGTTACTGAT-3′	5′-ACAGGACCGAAACATTCTGG-3′
*FN1*	5′-AGTGGGAGACCTCGAGAAGA-3′	5′-ACTGTGACAGCAGGAGCA-3′
*GAPDH*	5′-GAGTCAACGGATTTGGTCGT-3′	5′-GACAAGCTTCCCGTTCTCAG-3′

## Data Availability

Not applicable.
